# Prevalence of *Cryptosporidium* species among HIV positive asymptomatic and symptomatic immigrant population in Kashmir, India

**Published:** 2012-03

**Authors:** S Masarat, F Ahmad, M Chisti, S Hamid, B Ahmad Sofi

**Affiliations:** 1 Department of Zoology, S.P. College, M.A. Road, Srinagar; 2 P.G. Department of Zoology, University of Kashmir; 3 ART centre SKIMS, Hospital, Srinagar; 4 Department of Microbiology, SKIMS, Hospital, Srinagar

**Keywords:** Cryptosporidium, HIV-Infection, Asymptomatic and Symptomatic, immigrants, emigrants

## Abstract

**Background and Objectives:**

Cryptosporidiosis has not been reported as an endemic disease in Kashmir, but high prevalence of *Cryptosporidium* sp. has been found among asymptomatic (non-diarrheic) HIV positive immigrants in present study. Due to increasing number of HIV positive immigrants in Kashmir, *Cryptosporidium* may become a public health problem in Kashmir.

**Materials and Methods:**

A total of 45 stool samples were obtained from symptomatic (diarrheic n = 9) and asymptomatic (non-diarrheic n = 36) patients infected with HIV. The stool samples were concentrated using formalin ethyl acetate concentration technique, stained with modified Kinyoun's cold stain and oocysts were identified by microscopy under 1000 x magnification. It was confirmed by detection of antigens in stool samples by ELISA.

**Results:**

It was established that all the patients studied were carriers of *Cryptosporidium*. In present study though 80% of patients were asymptomatic (non-diarrheic) and HIV positive which involved non-Kashmiri army personals and travelers (immigrants) but were carriers of *Cryptosporidium* and 20% of HIV positive patients were emigrants (local Kashmiri traders) who travelled different states of India were having diarrhea (symptomatic) as well as carrier of *Cryptosporidium*.

**Conclusion:**

Though *Cryptosporidium* infection causes chronic diarrhea but in present study all HIV positive patients screened whether diarrheic or non-diarrheic were positive for *Cryptosporidium*. To prevent the transmission of *Cryptosporidium* oocyst in environment and endemic spread of cryptosporidiosis as non-diarrheic HIV positive population may be potential source of infection, obligatory laboratory testing for *Cryptosporidium* in HIV positive immigrant population like traders and travelers is highly recommended in order to have a better understanding of the cause of spread *Cryptosporidium* infection in Kashmir.

## INTRODUCTION

*Cryptosporidium*, an intracellular protozoan has changed from that of a rare largely asymptomatic disease, to an important cause of diarrhea in animals and humans worldwide and the potential for significant morbidity and mortality (1). Lately, the strong association between cases of cryptosporidiosis and immune-deficient individuals (such as those with AIDS) brought *Cryptosporidium* to the forefront as ubiquitous human pathogen (2). Reported Cryptosporidiosis prevalence is 3-4% in the USA (3), 3.5-22.4% in Brazil (4) and about 50% in Africa and Haiti (5). Reported prevalence of *Cryptosporidium* infection in Asia range from 3.6% in China (6) and 4.3% in Bangladesh (7). In India, there have been reports from the mid 1990s on the prevalence of cryptosporidiosis from different parts of the country ranging from 8.5 (8) to 81 per cent (9) with a high prevalence being reported from the north eastern States (9).

Presently, the increasing population of immuno-compromized persons and various outbreaks through infection by waterborne *Cryptosporidium* oocysts (often in drinking water) have placed an even greater emphasis on this pathogen.

*Cryptosporidium*, a small, obligate intracellular parasite, has emerged as an important cause of chronic life-threatening diarrhea (10); causing prolonged and cholera-like diarrhea in HIV infected patients (11). However, in immune-competent persons, it may cause a short-term diarrheal illness that resolves spontaneously. The route of transmission has been associated with fecal-oral, as well as through drinking contaminated water, person to-person spread and contact with infected animals (12).

A prospective long-term study from Europe suggested that 3-4% of those with HIV have cryptosporidiosis and that an equal number develop, it later in the course of their disease (13). However, this parasite has been identified in up to 46% of HIV patients world-wide in other studies (14). *Cryptosporidium* subtypes, genotypes and its molecular characterization has been studied (15–18). The global distribution of *Cryptosporidium* pathogen has been studied (19).

Opportunistic infections, to which HIV infected patients are susceptible, comprise only a minority of the large number of parasites capable of causing human diseases. Intestinal parasites that have exploited the immunological defect in HIV infected patients include *Cryptosporidium* species (20, 21).

In Kashmir, the prevalence of *Cryptosporidium* among HIV positive patients has not received much attention, as evidenced by the lack of reports. The increasing number of immigrants as army personals and travelers has brought about changes in social structure of Kashmir. The purpose of this study was to asses the prevalence of *Cryptosporidium* sp in HIV infected both diarrheic and non-diarrheic immigrant population in Kashmir by conventional staining and ELISA techniques which were available and to evaluate the risk factors. As there are no reports of cryptosporidiosis as an endemic disease in Kashmir but high prevalence rates submitted to this study, might indicate high risk of infection in the population, and may therefore be a public health concern. The objective of this study was to determine the prevalence of *Cryptosporidium* in HIV positive population especially non-diarrheic cases which may be source of infection for the spread of cryptosporidiosis, so that its early diagnosis and treatment is possible. The presence of parasite was confirmed by using both acid fast staining and ELISA techniques which were available.

## MATERIALS AND METHODS

### Study population

The present study was conducted only on HIV positive subjects and total of 45 stool samples were collected from patients which were found HIV positive both diarrheic (symptomatic n = 09) and non-diarrheic (asymptomatic n = 36) from antiretroviral therapy centre Sheri Kashmir Institute of Medical Sciences, Hospital, Srinagar, Kashmir, India with the permission of authorities and consent of patients. The patients complaining of gastro-intestinal illness but HIV negative were not included in this study as criteria selected were HIV positive. The patients examined included non-Kashmiri army personals and travelers (immigrants from different states of India) who stayed in valley for more than six months and local Kashmiri traders (emigrants) who visited different states of India and duration of their stay was again few months. The consistency of the stool specimens was graded by categories (soft, watery, etc) while waiting to be processed. The fresh stool samples were collected in sterile wide mouth containers. Each specimen was labelled, containing information about patient's name, age of patient and brief clinical history.

### Microscopic examination

All specimens were concentrated by the formalin-ethyl acetate method and stained with modified Kinyoun's acid fast stain. Stool specimen were labeled positive if oocysts between 4-6µm (as measured with an eyepiece micrometer) with typical morphology were identified by acid-fast stain.

### ELISA

The *Cryptosporidium* antigen in sample was detected by micro-well ELISA. The ELISA was performed with a commercially available kit according to the packaged instructions (Cryptosporidium Antigen Detection Microwell ELISA, Research, Inc. Carlsbad, CA 92010). The ELISA was performed on un-concentrated stools with a commercially available kit. Wash buffer provided with kit was diluted and 1gm of fresh or fresh-frozen stool samples were diluted in 3 ml of wash-dilution buffer and centrifuged at 500 x g for 10 min prior to testing. The required number of wells were broken and placed in holder. 100 µl of negative and 100 µl of positive control was added to first two wells and 100 µl of stool supernatant was added to each test well coated with *Cryptosporidium* polyclonal antibody, incubated with anti-goat *Cryptosporidium*, antibody conjugated to peroxidase, washed and then chromogen tetramethylbenzidine and peroxide was added to develop reaction. It was incubated for 5 minutes to develop blue color and reaction was stopped with addition of phosphoric acid, which changes blue color to yellow. The results were interpreted visually and by ELISA reader. The results were read visually in accordance with kit instructions, and an assay was considered valid if the control wells were appropriately positive and negative. The ELISA results were interpreted through use of a color shift indicating the presence of soluble antigen. The color change was assessed spectophotometrically measuring the optical density (OD). On the basis of data available from the Kit manufacturer absorbance reading of 0.15 OD units and above indicate that the sample contains *Cryptosporidium* antigen.

### Statistical analysis

The prevalence was calculated as:

$$\frac{\text{Total number of infected samples}}{\text{Total number of samples examined}}\times 100$$

The chi-square test was used to determine the relationship between the presence of *Cryptosporidium* oocysts in the patients who provided the stool samples, and other parameters such as diarrhea symptoms, and age and the P value < 0.05 was considered significant.

## RESULTS

Of the 45 samples analyzed during period of one year, all the samples of HIV positive patients were infected with *Cryptosporidium* infection. Since the purpose of present study was to determine the prevalence of *Cryptosporidium* in only HIV positive subjects, so samples of only HIV positive patients were screened and HIV negative patients were not included in study. Out of 45 samples, 36 (80%) were from immigrants- army personals and travelers from different parts of India who were HIV positive but non-diarrheic (asymptomatic) while 9 (20%) were from emigrants-local traders who visited different states of India and were HIV positive as well as diarrheic (symptomatic).

The sample was considered positive if organism could be detected both by acid fast staining and ELISA. The oocysts of *Cryptosporidium* were detected by acid fast staining in stool specimen and the presence of *Cryptosporidium* was confirmed by detection of its antigen in stool by ELISA. The range of patient's ages was 20-59 years. They consisted of 43 (95.5%) males and 2 (4.5%) females. These patients were travelers who stayed for few months outside the state and army personals who have been transferred to this state. The patients were grouped on basis of age, gender and symptoms in Table 1.

**Table 1:** Prevalence of Cryptosporidium infection in HIV positive patients with their age and sex distribution (n = 45).

Age group	Male	Female	Total	Diarrheic		Non-Diarrheic	
				No	%	No	%
20-29	07	2	9	2	22.2	7	77.7
30-39	26	0	26	4	15.3	22	84.6
40-59	10	0	10	3	30	7	70
**Total**	43	2	45	9	20	36	80

Our results showed that non-diarrheic immigrants had a significantly higher prevalence of *Cryptosporidium* infection than locals (80% vs. 20%, P < 0.001). *Cryptosporidium* was found at all ages as patients were selected randomly but the highest number of patients reported during this study belongs to age group 30-39 years. Statistical analysis showed that *Cryptosporidium* infection rates were significantly higher in non-diarrheic HIV positive patients aged between 30-39 years 84.6% than in those of age group 40-59 years and 20-29 (Table 1). Oocysts of *Cryptosporidium* sp. were found in stool samples but no other parasite stages could be detected in stool samples. Though *Cryptosporidium* infection causes diarrhea, but in present study highest number of patients were non-diarrheic (asymptomatic) but carriers of parasites.

## DISCUSSION

In this study, *Cryptosporidium* infection was the most common intestinal parasite among HIV positive non-diarrheic (asymptomatic) immigrant and emigrant patients who were either admitted or visited Sheri Kashmir Institute of Medical Science, Hospital, Srinagar, Kashmir, India. These immigrants may be source of infection as due to disposal of wastes in the river water; the locals may be predisposed to infection. *Cryptosporidium* is a major enteric pathogen of patients with acquired immunodeficiency syndrome (AIDS), with infection rates of 8–48% reported among African AIDS patients with diarrhea (5, 22–24). It has also been reported elsewhere (20, 21). Studies of patients with cryptosporidiosis indicate that a majority experience chronic diarrhea, while less than 15% have transient diarrhea or are asymptomatic (25, 26). The most important new finding in this study was the high rate of asymptomatic *Cryptosporidium* infection. The 80% rate of asymptomatic *Cryptosporidium* carriage in this study is higher than the 1–5% asymptomatic rate previously reported in AIDS infected patients from many developing world sites (22, 27).

Our study showed high *Cryptosporidium* oocysts count in fecal samples. The persons excreting oocysts of *Cryptosporidium* through their faeces were not complaining of any specific clinical symptoms such as diarrhea. Therefore most of the people that were positive for *Cryptosporidium* oocysts seemed to be in the carrier status of cryptosporidiosis and played a role of an infection source to the community.

The prevalence of *Cryptosporidium* infection among asymptomatic patients is in concordance with reports elsewhere; high rates of asymptomatic carriage (10–30%) are common in non-industrialized countries (28), 63% in Peruvian children (29) and 50% in Tanzania (30). High occurrences (23%) of *Cryptosporidium* in asymptomatic HIV positive IVDU drug users have been reported (31). The occurrence of asymptomatic carriers of *Cryptosporidium* has also been found to be common among AIDS patients (32). The presence of *Cryptosporidium* oocysts in asymptomatic patients is noteworthy because they can act as important reservoirs for the organism and might be a potential source of infection. It would be advisable to recognize that cryptosporidiosis can present with just chronic weight loss and other nonspecific symptoms devoid of diarrhea. Hence, a higher index of suspicion for clinical cryptosporidiosis in HIV patients, including those with chronic weight loss with or without diarrhea, is recommended. Additionally, laboratory testing for *Cryptosporidium* in HIV infected patients is highly recommended in order to have a better understanding of the epidemiology and management.

In Kashmir prevalence of *Cryptosporidium* have not been reported, so the high prevalence of *Cryptosporidium* infection among immigrants may pose a threat to local parasite free population. Status of *Cryptosporidium* in HIV positive population in Kashmir has been studied for the first time from this part of India and there is possibility of spread of this parasite in environment. For prevention of local transmission or endemic spread in Kashmir, routine health screening of immigrant population and early eradication should be important policies for high risk groups.

In conclusion, although the pathogenicity of *Cryptosporidium* infection requires further study, high prevalence of *Cryptosporidium* infection among non-diarrheic immigrants has also been observed. Our study was, therefore aimed at determining the prevalence and contribution of *Cryptosporidium* among HIV infected patients, and that asymptomatic shedding of oocyst in HIV positive patients may be probably important in transmission of disease, the knowledge of which will help in management of HIV related opportunistic infections in our environment.
